# Profile of Patients Suspected to be COVID-19: A Retrospective Analysis of Early Pandemic Data

**DOI:** 10.7759/cureus.10125

**Published:** 2020-08-29

**Authors:** Ashish Goel, Alpana Raizada, Kamakshi Bansal, Nikhil Gaur, Jyotika Abraham, Anil Yadav

**Affiliations:** 1 Internal Medicine, University Respati Yogyakarta, Yogyakarta, IDN; 2 Internal Medicine, University College of Medical Sciences, Delhi, IND; 3 Nursing, Guru Teg Bahadur Hospital, Delhi, IND; 4 Internal Medicine, Guru Teg Bahadur Hospital, Delhi, IND

**Keywords:** coronavirus disease 2019 (covid-19), reverse transcription polymerase chain reaction (rt-pcr)

## Abstract

Background and Objectives

Coronavirus disease 2019 (COVID-19), a global public health emergency of profound magnitude, has brought life to an unprecedented near-standstill. The clinical profile of the disease is still emerging and is marked by considerable geographical variability in terms of transmissibility, clinical profile, virulence, and mortality of the disease. As clinical data is being reported from around the globe, it becomes important to focus on local subjects in a global milieu, lest one misses the trees for the forest.

Our study is a short retrospective analysis of the demographic and clinical profiles of subjects presenting with a mild flu-like illness to our hospital who were tested for COVID-19. It compares the differences in age and sex of those who tested positive with those negative. In addition, it reviews the length of time it might take for a case testing positive on reverse transcriptase-polymerase chain reaction (RT-PCR) test to become negative.

Methodology

A retrospective analysis of data from adults who presented to our hospital with a mild flu-like illness between the months of March and May 2020 was conducted to understand the disease profile. The nasal/oropharyngeal swabs were collected from each patient and were transported to state-approved laboratories chain for RT-PCR analysis. Information was collected from reports received, clinical information forms, and sample collection forms that were being maintained as a part of the clinical management protocol. Data were analysed using Stata software, version 13 (StataCorp LLC, College Station, TX, USA).

Observations and Results

Three thousand twenty-six subjects presented to our hospital with either mild flu-like symptoms or with suspected exposure to a confirmed case of COVID-19. The subjects had a mean age of 37.3 (± 15.1) years and 1,805 (60.3%) were males. A regression analysis revealed an adjusted odds of 1.6 (95% confidence interval (CI): 1.2, 2.1) for testing positive for males as compared to females. For every one year increase in age, the odds for testing positive increased by 1.02 (95% CI: 1.01, 1.03).

Of the 2,592 individuals for whom data was available, 201 (7.6%) were found positive on RT-PCR analysis. Those testing positive were significantly older (41.0 years vs 36.8 years; p = 0.001) and more likely to be male (number: 138; 9.0% vs 6.7%; p = 0.05). Cough, followed by fever, was a common presenting feature.

A survival time analysis using data from 54 participants documented 455 days of the total observation period. A median time of eight days was required for the test to convert from positive to negative if the patient remained mildly symptomatic and did not develop a severe complicated illness. The time to conversion did not differ with age or sex.

Conclusions

Our analysis shows that patients with COVID-19 have presented with milder symptoms and have recovered well. The low test positivity rate is indicative of the early phase of the pandemic in the country and is a reflection of active infection control measures.

## Introduction

Coronaviruses, a family of viruses that range from the common cold to the coronavirus disease 2019 (COVID-19) and include the Middle East respiratory syndrome (MERS) coronavirus and the severe acute respiratory syndrome (SARS) coronavirus, has rapidly become a common household name in the space of a few months [1]. COVID-19 was declared a global public health emergency and has brought life to an unprecedented near-standstill owing to an unexpected, practically global lockdown. While comparisons have been drawn to the Spanish flu pandemic of 1918, the public health interventions in the present pandemic have been both brisk and far more severe.

Being an entirely new virus, a clinical profile of the disease is now emerging and has been characterized largely from Chinese studies [2-5]. As COVID-19 paves its way into India (there are over 178,014 active cases at the time of writing this paper), the disease dynamics in a naïve Indian population are only now being elaborated [6-7]. Considerable geographical variability has been noticed in the transmissibility, clinical profile, virulence, and mortality of COVID-19. Consequently, the COVID-19 dashboard, maintained by the Johns Hopkins Bloomberg School, indicates that while the case fatality rate in the United Kingdom is 13.9%, Australia has managed to escape with 1.4% [8]. India has had just over 14,000 deaths with the case fatality rate consistently under 3.5%. When the differences are so wide and cannot be explained by the preparedness of healthcare systems alone, it becomes important to explore host-characteristics. As clinical data emerge from areas outside of China, it becomes important to focus on local subjects in a global milieu, lest one misses the trees for the forest [9-11]. A recent case series of 21 patients from Delhi answers some questions but also raises several more unanswered questions [7].

We present a short retrospective analysis of the demographic and clinical profiles of subjects presenting with a mild flu-like illness to our hospital who were tested for COVID-19. It compares the differences in age and sex of those who tested positive with those negative. In addition, it reviews the length of time it might take for a case testing positive on reverse transcriptase-polymerase chain reaction (RT-PCR) test to become negative.

## Materials and methods

The proposal was reviewed by the Institutional Ethics Committee-Human Research (IEC-HR), University College of Medical Sciences, University of Delhi, Delhi (#IEC-HR/2020/44/2R), and a waiver of consent was approved for the present work.

A retrospective analysis of data from subjects who presented to our hospital with mild flu-like illness between the months of March and May 2020 was conducted to understand the disease profile. Data from adults who presented to our hospital (a non-COVID-designated facility during March through May 2020) in Delhi were included. Nasal/oropharyngeal swabs were collected from each patient and were transported to state-approved laboratories, maintaining the appropriate cold chain for RT-PCR analysis. 

Information was collected from the reports received, clinical information forms, and sample collection forms that were being maintained as a part of the clinical management protocol. Data were analyzed using Stata software, version 13 (StataCorp LLC, College Station, Texas, USA). Differences in proportions by groups (sex, clinical symptoms) were tested by using chi-square tests and the differences in means for age were examined using the t-test. The data was set-up as a survival time analysis to determine the median duration required for conversion from a positive to a negative test.

## Results

Data were available for 3,026 subjects who presented to our hospital with either mild flu-like symptoms or with suspected exposure to a confirmed case of COVID-19 during the early phases of the pandemic. The subjects had a mean age of 37.3 (± 15.1) years and 1,805 (60.3%) were males. The mean age was similar in males and females. A regression analysis revealed an adjusted odds of 1.6 (95% confidence interval (CI): 1.2, 2.1) for testing positive for males as compared to females. Every one year increase in age increased the odds for testing positive by 1.02 (95% CI: 1.01, 1.03)

**Figure 1:**
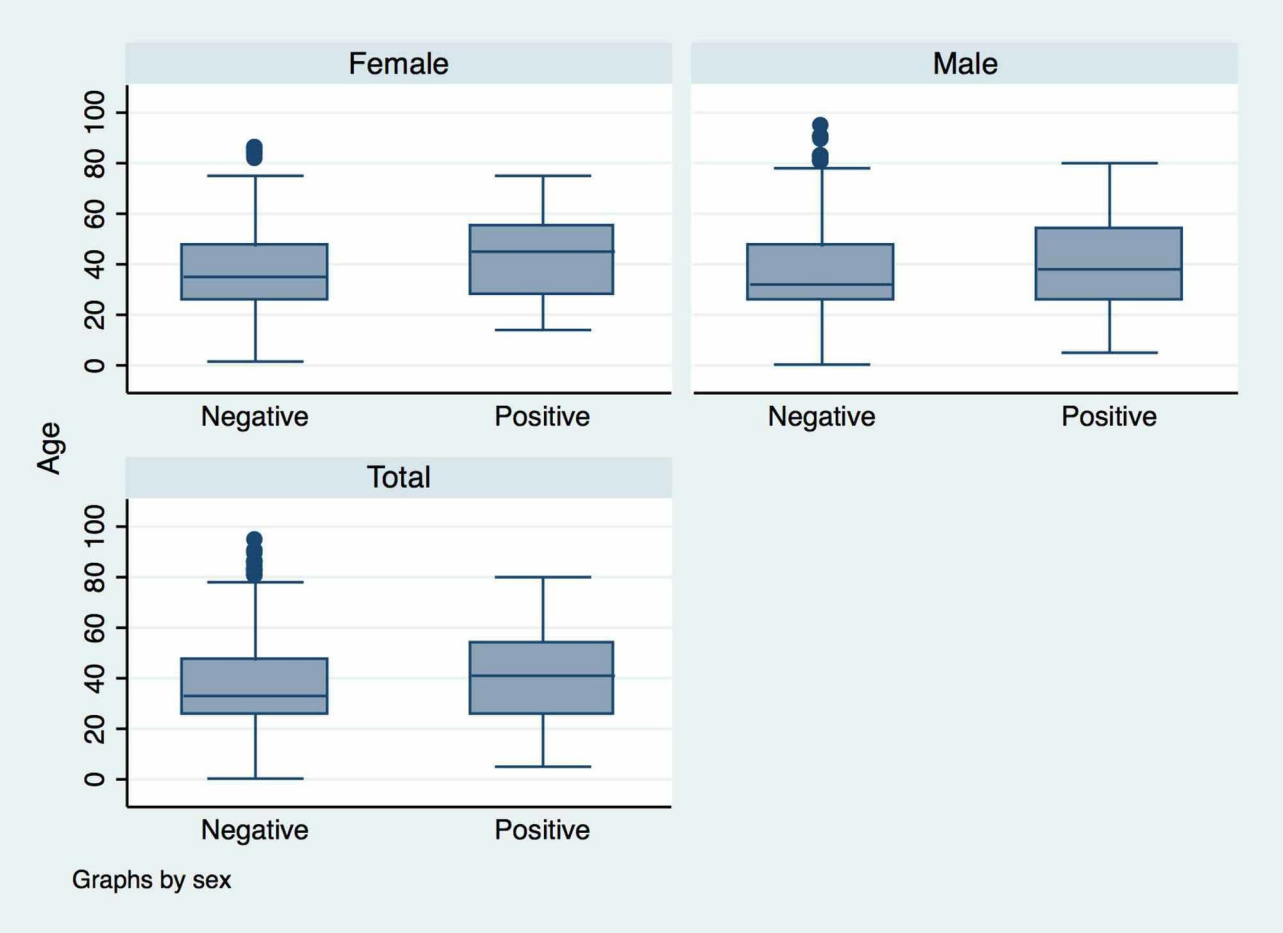
Boxplot showing the distribution of age over the test report

Of the 2,592 individuals on whom data was available, 201 (7.6%) were found to be positive on RT-PCR analysis and reports were inconclusive for 12 (0.5%). Those testing positive were significantly older (41.0 years vs 36.8 years; p = 0.001) and more likely to be male (n: 138, 9.0% vs 6.7%; p = 0.05). The age distribution of patients based on the test report is presented in Figure 1. 

Limited data were available on the clinical profile of 31 patients who had tested positive. The mean age of this group was 39.2 years (± 16.7) and there were 25 (80.1%) males. Only one patient had symptoms for more than a week. Most individuals who were found to be positive presented in the first week of illness. Among these 31 patients who tested positive, the commonest presenting features were cough (seen in 16 patients, 51%), fever (seen in eight patients, 26%), myalgia (seen in three patients, 10%), and breathlessness (seen in three patients, 10%) with some overlap between symptoms. Ten patients who were found to be positive did not have a clear history of exposure at presentation. 

**Figure 2:**
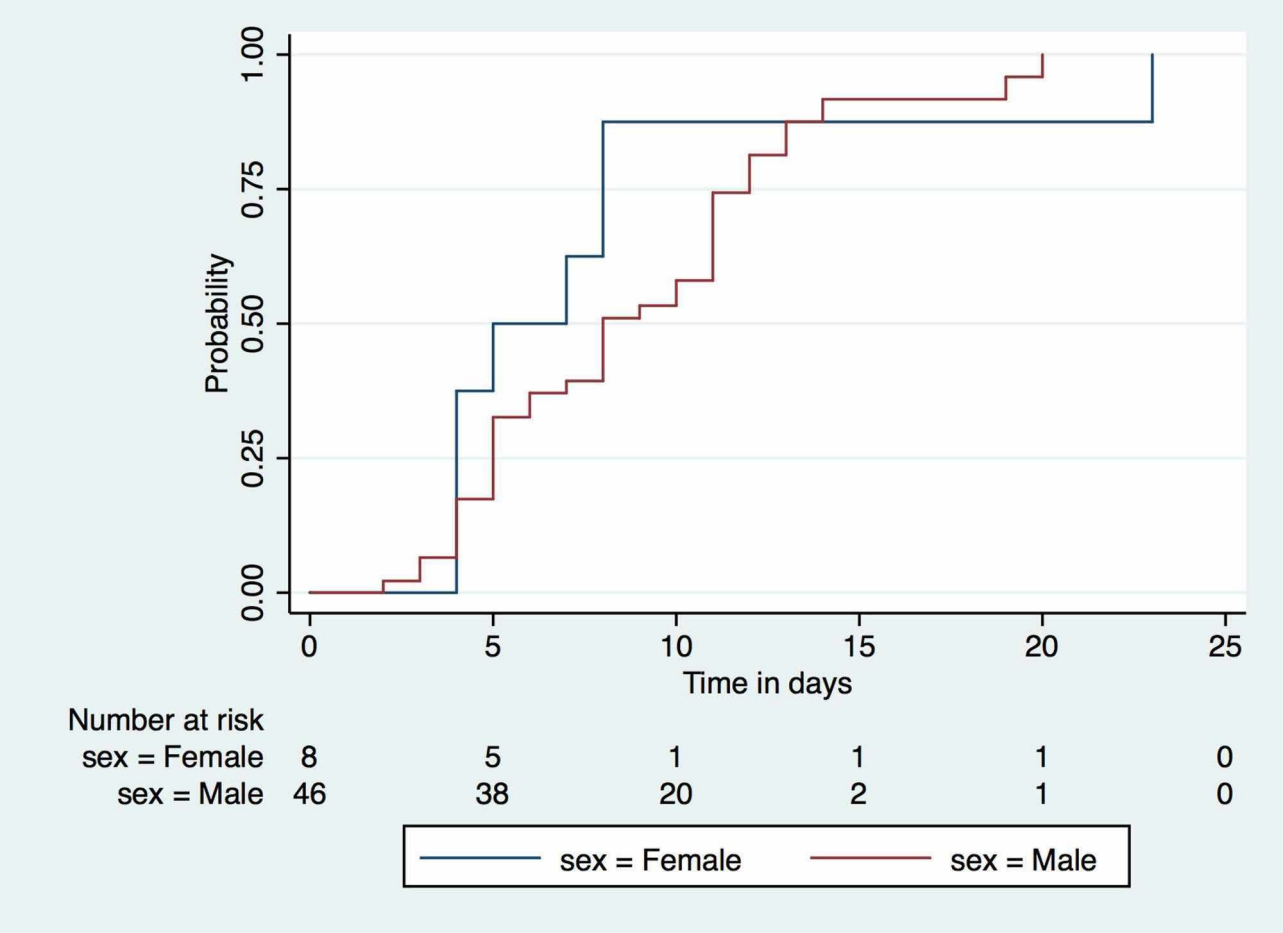
Kaplan-Meier estimate showing time to recovery after testing positive

A survival time analysis was conducted, including available data from 54 patients who contributed 455 days of the total observation period. Once a patient tested positive for COVID-19, it took a median time of eight days (interquartile range (IQR): 5, 11 days) for the test to become negative if the patient remained mildly symptomatic and did not develop a severe complicated illness. The maximum time to test negative was 23 days over a total observation time of 455 days contributed by a total of 54 patients. The time to conversion did not differ with age or sex as shown in Figure 2.

## Discussion

In this retrospective analysis, we report that among subjects presenting to the hospital with a mild flu-like illness, those who tested positive for COVID-19 were significantly older and more likely to be men. Patients who tested positive took a median of eight days to become negative for COVID-19 by RT-PCR.

In a retrospective study by Xiao et al. of 301 confirmed COVID-19 patients hospitalized at Tongji Hospital in Wuhan, China, the median age was 58 years and 51.2% were male [12]. The median period between onset of symptoms and positive severe acute respiratory syndrome coronavirus-2 (SARS-CoV-2) RT-PCR results was 16 days (IQR: 10 - 23, n = 301). The median period of RT-PCR results to turn negative was 20 days (IQR: 17 - 24; n = 216). Infected patients ≥ 65 years old stayed contagious longer (22 days vs 19 days, p = 0.015).

In another study conducted by Liu et al. at Wuhan in the early days of the pandemic, 4,880 cases were tested for SARS-CoV-2 [13]. The median age was 50 years (IQR: 27) with 46.13% of the subjects being male. Out of the 4,880 patients, 1,875 (38.42%) were positive by RT-PCR. Males had a significantly higher positivity rate than females (40.43% vs 36.71%, respectively; p < 0.01) [13]. 

Escalera-Antezana et al. evaluated 152 subjects in a retrospective cross-sectional analysis and found that 12 (7.9%) of them returned positive results by real-time reverse-transcriptase polymerase chain reaction (rRT-PCR) [14]. The median age of the infected subjects was 39 years (IQR: 25 - 43) and half of them were male. Most subjects remained mildly symptomatic and all recovered. 

The earliest data to emerge from across the world have consistently described patients who have presented with a milder illness and have rapidly recovered [9, 15-16]. Kim et al. observed 213 COVID-19 patients in South Korea and found that 19% remained asymptomatic [14]. Others had a cough (40%) and hypoxia (40%). Joshi et al. described nine relatively younger men in a small case series from Nepal who had a mean age of 20 - 40 years [15]. 

In a retrospective analysis of 95 patients hospitalized during three weeks in March in the United Kingdom, Tomlins et al. reported that less than half of them could be discharged and one in five patients died [17]. They reported that diabetes, cerebrovascular, and cardiovascular illness were associated with a poorer outcome. Most of their patients presented with fever and cough, while anosmia was under-represented. Those presenting with breathlessness had a greater chance of dying. While mortality appears to be considerably higher in their study as compared to other international reports, it is noteworthy that their patients were older (mean: 75 years).

Goyal et al. retrospectively analysed data from 393 patients, with a mean age of 62 years, hospitalized in New York with common symptoms of cough, fever, and breathlessness [11]. Forty patients (10.2%) died, and obese men had more severe disease.

Colaneri et al. from Italy retrospectively studied 44 COVID-19-positive patients and nearly 39% of the patients in their study developed a serious illness, while two (4.5%) died [10].

Closer home, in a series of 21 case studies, Gupta et al. documented a mean age of 40.3 years with male preponderance (66.7%) [7]. The most common clinical presentation was cough and fever. However, 43.9% of individuals were asymptomatic. Hypertension, followed by diabetes, was the most common comorbidity seen.

The COVID-19 patients in India are younger as compared to their Western counterparts. Male preponderance has been seen worldwide. The proportion of asymptomatic individuals seems to be substantial and may be attributable to active contact tracing and surveillance in the country. Time taken to document COVID-19 negativity by RT-PCR in our study is lesser and may be explained by the relatively younger age composition in our study. However, we did not find any difference in time to negativity based on age.

The test positivity rate of 7.6% represents the early phase of the pandemic in India and is also a marker of the prompt availability of free testing facilities and active surveillance by the government. 

The present study is the first analysis of structured patient data inclusive of those who tested negative for COVID-19 from India. The study can be used as a baseline to assess changes in test positivity rates overtime that may show the transition of the pandemic from one phase to another. The study provides important inputs for informed decision-making in patient management and helps in understanding the local trend of the disease.

There is limited data available since, during the early part of the pandemic, data collection and electronic registries were not as robust, leading to gaps in information.

## Conclusions

Our analysis shows that patients with COVID-19 have presented with milder symptoms and have recovered well. Of the individuals who were tested for COVID-19, 7.6% were found to be COVID-19 positive and most patients recovered after a mild illness. If global trends are to be believed, the pandemic could still be in its early stages in Delhi and the numbers have remained low due to the early, brisk, and effective public health measures taken by the State. However, there is little room for complacence and the disease may take a more severe form in the not so distant future, as has been sadly realized by other countries.
